# Yeast Particles Hyper-Loaded with Terpenes for Biocide Applications

**DOI:** 10.3390/molecules27113580

**Published:** 2022-06-02

**Authors:** Ernesto R. Soto, Florentina Rus, Gary R. Ostroff

**Affiliations:** Program in Molecular Medicine, University of Massachusetts Medical School, Worcester, MA 01605, USA; ernesto.soto-villatoro@umassmed.edu (E.R.S.); florentina.rus@umassmed.edu (F.R.)

**Keywords:** yeast particles, terpenes, microencapsulation, biocide, controlled delivery

## Abstract

Yeast particles (YPs) are 3–5 µm hollow and porous microspheres, a byproduct of some food grade yeast (*Saccharomyces cerevisiae*) extract manufacturing processes. Terpenes can be efficiently encapsulated inside YPs by passive diffusion through the porous cell walls. As previously published, this YP terpene encapsulation approach has been successfully implemented (1) to develop and commercialize fungicide and nematicide products for agricultural applications, (2) to co-load high potency agrochemical actives dissolved in terpenes or suitable solvents, and (3) to identify YP terpenes with broad-acting anthelmintic activity for potential pharmaceutical applications. These first-generation YP terpene materials were developed with a <2:1 terpene: YP weight ratio. Here we report methods to increase the terpene loading capacity in YPs up to 5:1 terpene: YP weight ratio. Hyper-loaded YP terpenes extend the kinetics of payload release up to three-fold compared to the commercialized YP terpene formulations. Hyper-loaded YP-terpene compositions were further optimized to achieve high terpene storage encapsulation stability from −20 °C to 54 °C. The development of hyper-loaded YP terpenes has a wide range of potential agricultural and pharmaceutical applications with terpenes and other compatible active substances that could benefit from a delivery system with a high payload loading capacity combined with increased payload stability and sustained release properties.

## 1. Introduction

Terpenes are a large class of naturally occurring compounds that constitute the primary components of essential oils obtained from plants. Terpenes have received great commercial interest due to their wide range of functional properties. Terpenes are used as permeation enhancers and antioxidants in cosmetics, for their broad range of potential anti-infective applications against pathogens in agricultural products, as additives in food packaging to prevent bacterial spoilage oxidation, and in pharmaceutical products as bioactive compounds or as excipients due to their permeation enhancer properties [1,2,3,4]. A potential benefit of terpenes in pharmaceutical applications is that the multiple mechanisms of action (e.g., interference with the phospholipid layer of cell membranes, blocking of cholesterol synthesis, impairment of enzyme systems) evolved over millennia as plant defense molecules make it extremely unlikely for infective agents to build resistance against terpenes [5,6]. Applications of terpenes will continue increasing to address consumer trends favoring the use of natural antimicrobials over chemical preservatives in food, agricultural, consumer, and pharmaceutical products.

There are, however, a few challenges in using terpenes in commercial products primarily associated with some terpene’s chemical stability and poor water solubility. Most terpenes are volatile oils and are prone to chemical degradation when exposed to air, heat, light, and moisture. It is usually necessary to prepare terpene-based products with high levels of surfactants or alcohols to protect terpenes and to increase their solubility in water. Terpene formulations that are produced as aqueous emulsions or dry powder formulations generally contain a low percentage of terpenes by weight. Applications of terpenes in certain products, such as food preservatives, pose additional challenges due to safety limits and marked organoleptic effects.

Encapsulation of terpenes in nano or micro-structured carriers is an innovative and promising approach to overcome the challenges of terpene instability, poor water solubility, and unacceptable taste/odor, as well as to potentially provide for controlled terpene release and targeted delivery [4,7]. Encapsulation techniques currently employed in the preparation of terpene products include emulsification, extrusion, fluidized bed coating, spray drying, liposomes, molecular inclusion, coacervation, in situ polymerization, and nanostructured lipid matrices [8,9,10,11,12]. A nano or microencapsulation process should (1) have high terpene encapsulation efficiency, (2) have high terpene loading capacity, (3) provide homogenous terpene distribution in the payload carrier, (4) be low cost and operate under mild processing conditions, (5) improve terpene protection, (6) possess sustained release characteristics, and (7) retain the biological activity of terpenes. The materials selected for terpene encapsulation should have mechanical strength as well as controlled release properties and be non-toxic. Commonly used materials for microencapsulation of terpenes include gum Arabic, gelatin, chitosan, modified starches, maltodextrin, and other materials that are Generally Recognized as Safe (GRAS) and therefore acceptable for applications in food, cosmetic, pharmaceutical, and agricultural products [4,11].

We have developed methods using yeast particles (YPs) to efficiently encapsulate high levels of terpenes. YPs are 3–5 μm hollow and porous microspheres, a byproduct of some food grade Baker’s yeast (*Saccharomyces cerevisiae*) extract manufacturing processes. We have used YPs for the encapsulation of a broad range of molecules for drug delivery and agricultural applications [13,14,15,16,17,18,19,20,21]. Terpenes can be loaded inside the hydrophobic cavity of YPs based on the passive diffusion of terpenes through the porous yeast cell walls. Sustained release from YPs is the reverse process and is a function of terpene-water solubility. This approach has been successfully implemented to develop and commercialize YP-terpene based fungicide and nematicide products for agricultural applications [22,23,24,25,26,27]. More recently, we have identified YP terpenes with broad-acting anthelmintic activity [20] which could lead to the development of formulations for oral terpene delivery for the treatment of gastrointestinal worm parasites and other infectious agents. In addition, we have developed a YP terpene encapsulation approach using non-volatile, biodegradable pro-terpene compounds for improved stability and controlled, sustained terpene release from YPs [19,21].

In this article, we report methods to increase the terpene loading capacity in YPs up to a 5:1 terpene: YP weight ratio. These hyper-loaded YP terpene formulations fully retain the terpene’s biological activity and extend the duration of payload release up to three-fold compared to the earlier generation YP-terpene (1.1:1 terpene: YP weight ratio) formulations used in commercialized products. The development of hyper-loaded YP terpenes has a wide range of potential agricultural and pharmaceutical applications that could benefit from a delivery system with high terpene loading capacity, payload stability, and sustained terpene release.

## 2. Results and Discussion

### 2.1. Preparation and Characterization of Yeast Particles Hyper-Loaded with Terpenes

**Figure 1:**
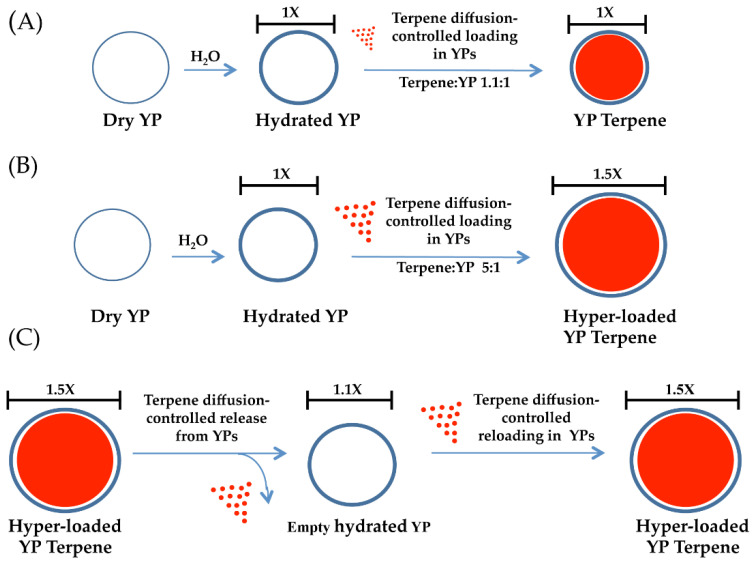
Schematics of diffusion-controlled terpene loading in YPs at terpene: YP weight ratios of (**A**) 1.1:1 and (**B**) 5:1, showing an increase in yeast particle diameter for hyper-loaded samples. Schematics of terpene release from hyper-loaded YPs and terpene re-loading and YP elasticity (**C**).

Terpenes are natural compounds of great commercial interest due to their wide range of functional flavor, fragrance, and biological properties (e.g., anthelmintic, antibacterial, antifungal, and antioxidant properties). However, the use of terpenes in some products is challenging due to terpene water solubility, volatility, and susceptibility to degradation. We previously developed methods to use 3–5 µm YPs for the encapsulation of terpenes or biodegradable pro-terpene compounds without the need for alcohol or surfactants [19,20,21]. YPs are 3–5 μm hollow and porous microparticles derived from Baker’s yeast and are composed primarily of ~80% 1→6-β branched, 1→3-β-glucan, 2–4% chitin, and 40% mannan *w*/*w* [28]. The porous cell wall structure makes these particles excellent absorbent materials, and payloads can be loaded from aqueous and some organic solutions with high payload loading capacity into the large hollow YP cavity. Terpene encapsulation in YPs is based on the loading of terpenes inside the hydrophobic cavity of YPs by the passive diffusion of the payload through the porous cell walls as depicted in Figure 1.

Here, we have evaluated the production of YP terpenes at terpene: YP weight ratios up to 5:1 using a mixture of three terpenes (geraniol, eugenol, and thymol or GET) at a composition of 2:1:2 G:E:T weight ratio. This GET composition has been proven to be highly effective in antifungal and antinematicidal agricultural applications against a broad range of plant pathogens [22]. The chemical structures, water/octanol partition coefficient, and solubility in water of the selected terpenes are shown in Table 1.

**Figure 2:**
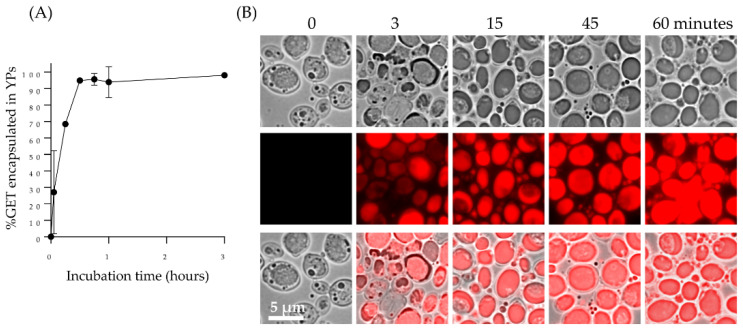
(**A**) Kinetics of 2:1:2 GET loading in YPs quantified by HPLC of samples prepared at GET:YP ratio of 1.1:1 in a homogenized YP suspension (final concentration of 150 g YP/L, 16.5% GET), and (**B**) microscopy images of Nile red-stained YP control (t = 0) and YPs loaded with GET collected at different timepoints showing Nile red-stained terpenes encapsulated in YPs.

The passive diffusion of the GET mixture into YPs in a homogenized aqueous YP suspension (GET:YP weight target ratio of 1.1:1) is a rapid process and >95% of the terpenes are encapsulated in YPs within one hour as shown by HPLC quantification of YP encapsulated GET (YP-GET) in Figure 2A and Supplementary Materials Figure S1. Nile red dye was used to stain terpenes to visualize payload encapsulation as shown in Figure 2B. Terpene encapsulation in YPs is achieved with high encapsulation efficiency and homogenous terpene distribution in the particles. Terpene encapsulation in YPs at a target terpene: YP ratio of 1.1:1 has been successfully implemented to develop and commercialize YP-terpene based fungicide and nematicide products for agricultural applications [22,23,24,25,26,27].

**Table 1:** Chemical structure, water/octanol partition coefficient (log P), and solubility in water of the terpenes used for loading in YPs.

Terpene	Log P *	Solubility in Water * (mg/mL)
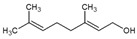 Geraniol	3.56	0.686
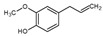 Eugenol	2.27	1.44
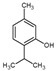 Thymol	3.3	0.9

* Log P and solubility data from Pubchem [29].

**Figure 3:**
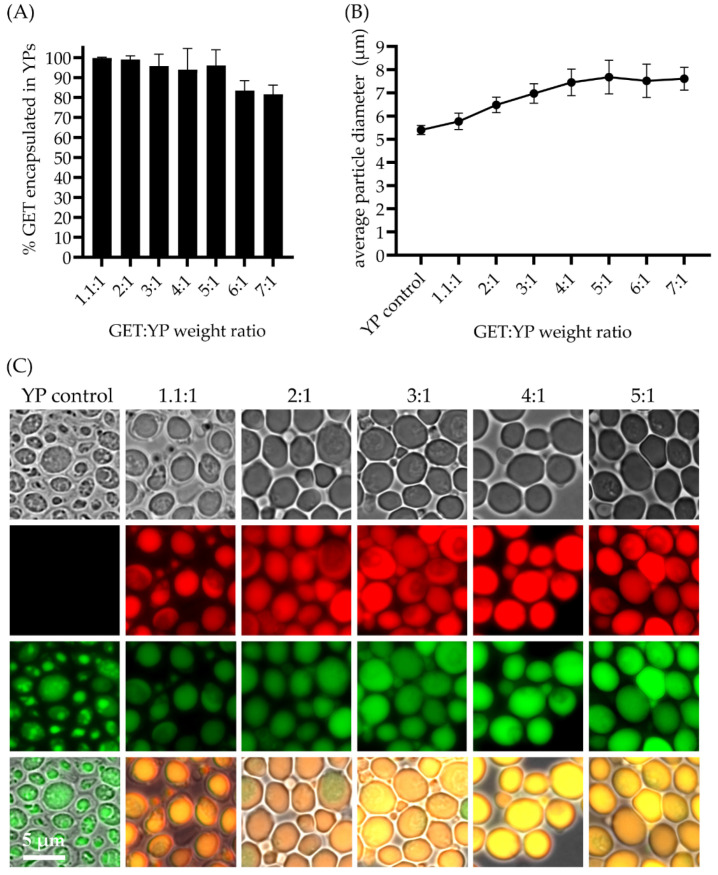
(**A**) Terpene encapsulation efficiency in YPs at GET:YP weight ratios from 1.1:1 up to 7:1 of samples prepared at 150 g YP/L (1.1:1 to 5:1 GET:YP) and 100 g YP/L (6:1 and 7:1 GET:YP), (**B**) average particle size of empty YP control and GET loaded YPs, and (**C**) microscopy pictures of control and GET loaded particles showing YP stained with FITC ConA and encapsulated terpenes stained with Nile red.

To improve the volumetric delivery of the original YP-GET 1.1:1 formulation, we have produced YP-GET formulations with up to five-fold higher terpene levels resulting in a three-fold increased sustained terpene release. YP-GET samples were prepared with GET:YP weight ratios up to 5:1 at a YP concentration of 150 g YP/L. The encapsulation efficiency results and microscopy images in Figure 3 show that terpenes can be encapsulated in YPs with >90% efficiency for all ratios up to 5:1. There is the possibility that terpenes could be in three locations: (1) as an oil droplet inside the hollow cavity of YPs, (2) trapped within the pores of the YP cell wall matrix, and (3) absorbed as a small amount of terpene on the surface. The leftover (<1%) is material that is soluble in water and is in equilibrium with the encapsulated terpenes in the YP GET materials produced as highly concentrated YP terpene aqueous suspensions. The hyper-loading of terpenes in YPs leads to an increase in YP size with an average particle diameter (diameter of the major axis of the YP ellipsoids) increase from 5.4 μm (empty YPs) to 7.7 μm (YP GET 5:1), as shown in Figure 3B. It is known that β-1,3-glucan chains are highly elastic because of their helical structure and that the inner β-1,3-glucan layer provides the majority of the mechanical strength of Baker’s yeast cell walls [30]. Although YPs contain chitin, which has an elastic modulus 10 times greater than other components of the cell wall, it is not essential for the stability and mechanical strength of Baker’s yeast [31,32]. The mechanical properties of Baker’s yeast cells have been studied by compression testing by micromanipulation, a technique that allows measuring properties such as the elastic modulus of particles with sizes as small as 1 µm. These studies have shown that Baker’s yeast cells possess an elastic modulus of 185 ± 15 MPa [33]. This elastic modulus value is consistent with a somewhat flexible material as shown by the ability of YPs to swell to accommodate for the inclusion of terpenes up to five times the weight of the particles (as a comparison, yeast cells have a modulus slightly higher than rubber (10–100 MPa) but more than five times lower than a less flexible bacteriophage capsid (1–3 GPa) [34].

The YP-GET results shown in Figure 2 and Figure 3 correspond to materials prepared at a YP final concentration of 150 g/L, terpene content from 165 up to 750 g/L, and water. It is critical for efficient loading in YPs to use water as the solvent as YPs swell very efficiently in water, allowing for pore opening and diffusion of the payload. A minimum volume of 0.5 μL water per 1 mg YPs is required for efficient loading therefore limiting the preparation of hyper-loaded YP terpenes to a maximum ratio of 5:1 at the target YP concentration of 150 g/L. We prepared YP-GET samples at ratios greater than 5:1 by mixing YP, water, and GET to generate samples with a final YP concentration of 100 g/L and GET:YP ratios of 6:1 and 7:1. The terpene encapsulation efficiency decreased to ~80% and there was no significant increase in particle diameter in these samples compared to the YP-GET 1:5 sample. These results show that the maximum loading capacity of GET in YPs is between 5:1 to 5.5:1 GET:YP weight ratio.

### 2.2. Terpene Release from Hyper-Loaded YP-GET

**Figure 4:**
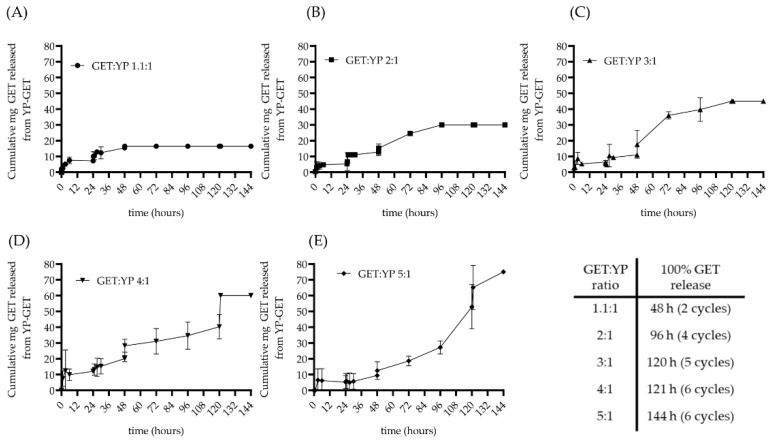
Cumulative GET release from YPs showing extension of wetting/terpene release cycles in hyper-loaded YP-GET samples: (**A**) GET:YP 1.1:1, (**B**) GET:YP 2:1, (**C**) GET:YP 3:1, (**D**) GET:YP 4:1, and (**E**) GET:YP 5:1.

Sustained terpene release from YPs occurs by terpene diffusion out of the particles and is a function of terpene solubility in water. The terpenes in the GET composition have maximum solubility in water of 0.686 (geraniol), 0.9 (thymol), and 1.44 mg/mL (eugenol). A 1:100 dilution of a YP-GET 1:1.1 (150 mg YP/mL, 165 mg GET/mL) generates a sample with a total GET concentration in water of 1.65 mg GET/mL. The concentration of each terpene is below its maximum solubility in water at this 1:100 dilution. The results in Figure 4A for YP-GET 1.1:1 show sustained release of ~7.5 mg (<50% GET content in a 100 mg YP-GET 1.1:1 sample) during the first 6 h of incubation and no additional release from 6 to 24 h. The plateau after six hours indicates that some GET is retained in the particles, likely due to interactions with hydrophobic lipids in the YP cell walls. A second dilution (1:1) was done at 24 h to achieve complete GET release from YP-GET 1.1:1.

The hyper-loaded YP-GET samples were evaluated with the same procedure starting with a constant amount of YP-GET (100 mg) diluted in 10 mL to generate samples of varying GET content from 16.5 mg up to 75 mg GET. The samples were diluted 1:1 every 24 h to determine the number of cycles to achieve complete GET release from YPs (Figure 4B–E). The GET release results show that (1) hyper-loaded YP-GET are stable, as no burst release leading to the release of an emulsion of terpenes in water and empty YPs was observed upon dilution, and (2) it is possible to extend the number of wetting/terpene release cycles three-fold from the two cycles for the commercialized YP GET 1.1:1 up to six cycles for the hyper-loaded YP GET 4:1 and 5:1 formulations. Extending the duration of terpene sustained release could significantly enhance efficacy and time between spraying for agricultural applications of YP-GET in soils, field crops, post-harvest decay, and seed treatments. The kinetics of terpene release from YPs was evaluated in water to simulate release conditions in rainwater and ambient humidity and in 0.9% saline to simulate groundwater and biological fluids (Supplementary Materials Figure S2). The salt concentration has no effect on terpene release.

The analysis of the GET released from the particles showed a similar release pattern for each terpene in each of the five YP GET samples indicating that the three terpenes are releasing together as an isotropic mixture and not differentially releasing based on their water solubility, as eugenol and thymol are significantly more water soluble than geraniol (release data for each terpene can be found in Supplementary Materials Figure S3). This is important for the consistent antimicrobial bioactivity of this mixture of terpenes released over time.

### 2.3. Biological Activity of Hyper-Loaded YP GET

The antimicrobial biological activity of YP-GET samples was evaluated against different model microbial organisms to show that YP-GET retains the broad-spectrum antimicrobial effects of free GET. Terpenes have strong membrane permeation properties, and a primary mode of action is the disruption in structural changes of the plasma membranes of both fungi and bacteria [35,36]. The lipophilic isoprene unit of terpenes exhibits great affinity for the lipid portion of plasma membranes and the hydrophilic polar groups increase this activity because of their interactions with proteins and carbohydrates [37,38].

The results in Table 2 show all YP-GET samples were active against *Escherichia coli*, *Staphylococcus aureus*, and *Candida albicans*, and GET loading in YPs appears to enhance terpene MIC. Generally, unencapsulated GET emulsions are less stable and are four-fold less potent than the YP encapsulated GET formulations. The *E. coli* and *S. aureus* bacteria require a much lower concentration of ampicillin, and *C. albicans* requires a 10-fold lower concentration of fluconazole than YP GET to reach MIC 75%. However, resistance to ampicillin and fluconazole is common, whereas attempts to isolate strains resistant to 10-fold higher concentrations of the GET monoterpenoids have repeatedly failed in our hands. Further, ampicillin-resistant and fluconazole-resistant bacterial and fungal strain susceptibility to YP-GET remains the same as the sensitive strains, showing the value of using monoterpenoids as a biocide (data not shown).

### 2.4. Optimization of Hyper-Loaded YP-GET Formulations as Dry Granules or Aqueous Suspension Concentrates

A drawback of hyper-loaded YP-GET samples prepared with a final composition of 15% *w*/*w* YP is that these samples exhibit poor flowability at ratios ≥ 3:1 GET:YP, preventing their application as aqueous suspension concentrates. The flow rate of a YP-GET 1:3 at 15% YP is 0.0003 cm^3^/s which represents an ~80% reduction compared to the flow rate of YP-GET 1.1:1 at 15% YP. Hyper-loaded YP-GET samples ≥ 3:1 GET:YP can be processed into dry YP-GET granules by an extrusion process or could be prepared at lower YP concentrations to improve the flow rate of the final product and applied as YP-GET aqueous suspension concentrates. We prepared samples at lower YP concentrations to improve the flow rate of the final product. YP-GET samples at 5% and 10% YP at GET:YP ratios of 3:1 and 4:1 were produced with high encapsulation efficiency (>95%) and the reduction of YP concentration generated samples with similar flowability to YP-GET 1.1:1 at 15% YP.

**Figure 5:**
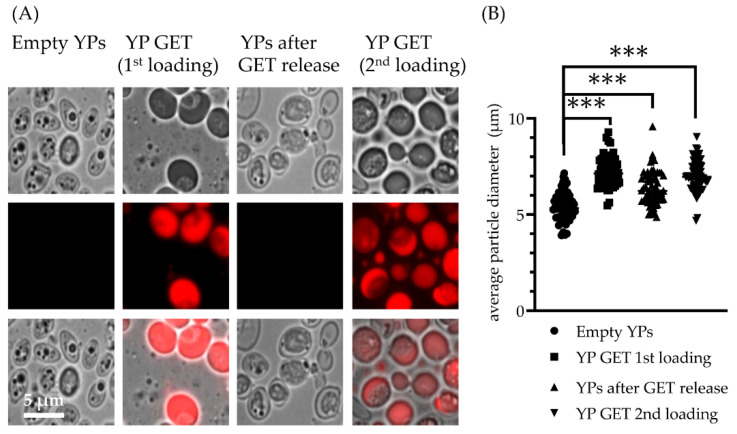
(**A**) Microscopy images of YP-GET 1:3 (10% YP, 30% GET) showing YPs remain intact following terpene loading, release, and terpene re-loading, and (**B**) average yeast particle diameter measured along the major axis of the particles (results show measurements of 60 particles per sample). Statistically significant results (*t*-test) were obtained between average particle diameter of empty YPs and YP GET samples (*** *p* < 0.001).

The optimized YP-GET samples with a composition of 10% YP and 30% GET were evaluated for GET release and the empty YP samples were loaded again with GET at the same ratio of 3:1. The schematic in Figure 1C depicts, and microscopy images in Figure 5 confirm, good encapsulation of GET after first loading, empty YPs after complete release of GET in five cycles, and efficient encapsulation of GET after second loading. The YP diameter measurements in Figure 5B show there is hysteresis following the release of hyper-loaded GET from YPs, as there was only a partial reduction of average particle diameter (YPs after the release of GET have an average diameter of 6.2 μm compared to the original YP average diameter of 5.4 μm).

**Figure 6:**
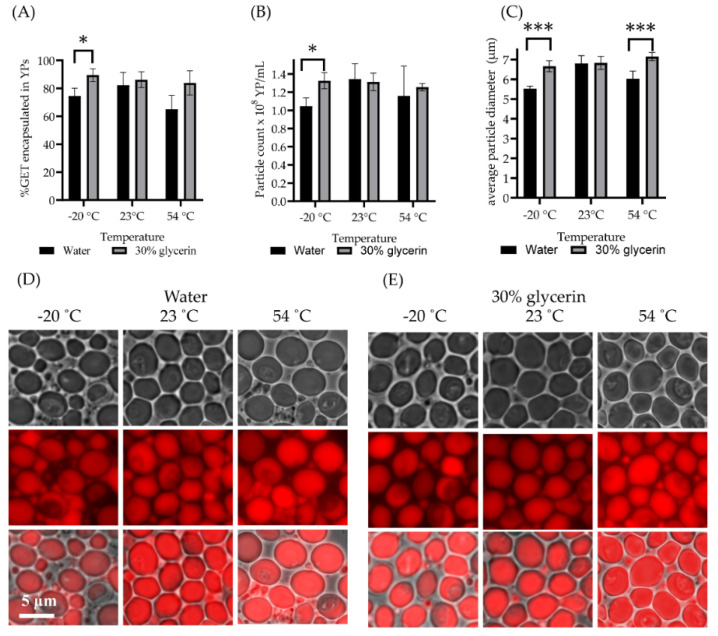
YP-GET (10% YP, GET:YP ratio of 3.5:1) prepared in water as loading solvent and 30% glycerin–70% water in stabilized YP-GET formulation. Results show (**A**) % GET encapsulation, (**B**) particle count, (**C**) average particle diameter, and (**D**,**E**) microscopy of samples after temperature stress (repeated freeze/thaw cycles and two-week storage at 23 °C and 54 °C). Statistically significant results were determined by *t*-test (* *p* < 0.1, *** *p* < 0.001).

Next, we evaluated the storage encapsulation stability under temperature stress conditions from −20 °C to 54 °C. The YP-GET samples were not stable showing between 15% to 40% GET release after storage at −20 °C and 54 °C at all GET:YP ratios. The major reason for instability was the appearance of broken shells prematurely releasing their terpene contents. We hypothesized that YP-GET could be stabilized for storage at low and high temperatures by the incorporation of a cryoprotectant during the loading of GET in YPs. Glycerin is a GRAS compound used as a cryoprotectant in biological applications, such as the low temperature storage of blood cells. Glycerin works as a cryoprotectant by forming strong hydrogen bonds with water. A mixture of 30% glycerin and 70% water has a freezing point of −38.9 °C and a boiling point of 114 °C [39] and therefore is a suitable solvent system to improve the temperature encapsulation stability of YP-GET. It is possible to prepare YP-GET samples using 30% glycerin at YP concentration of 100 g/L and GET:YP ratios of 3:1 up to 4.5:1. These samples were produced with high encapsulation efficiency (>90%), have the same MIC as YP-GET samples shown in Table 2, and the use of 30% glycerin as solvent did not impact GET release. We evaluated these stabilized YP-GET samples for storage encapsulation stability following temperature stress. The results in Figure 6 show the encapsulation stability results of YP-GET samples prepared in water and 30% glycerin–70% water. The results show that samples prepared in 30% glycerin are more stable at all three temperatures than the YP-GET samples without glycerin. YP-GET samples prepared in water show a reduction of particle number (Figure 6B) after storage at −20 °C due to particle breakage by ice crystals, and a reduction of the amount of encapsulated terpene (Figure 6A) and average YP diameter due to partial GET loss (Figure 6C) at both −20 °C and 54 °C.

The importance of achieving these high levels of terpene encapsulation in YPs include: (1) extended sustained terpene release, (2) eliminated the need for homogenization to produce single particle suspension concentrates, (3) reduced YP costs, while maintaining (4) terpene stability, and (5) enhanced bioactivity over free terpenes. The previously developed YP GET 1.1:1 formulation simplified handling and applications of terpenes in agricultural applications and the sustained release increases the duration of action of the terpene in the soil or on plants compared to free terpenes. The new hyper-loaded YP-GET samples extend the duration of terpene release up to three-fold compared to the commercialized YP GET 1.1:1 formulation. The methods developed to generate temperature-stable hyper-loaded YP-GET could be used for other YP single terpene or terpene mixtures. The development of these hyper-loaded YP terpenes has a wide range of potential applications offering high payload loading capacity, increased payload stability, and sustained release properties.

## 3. Materials and Methods

Yeast particles (YPs) were purchased from Biorigin (Louisville, KY, USA). Terpenes (eugenol, thymol, and geraniol) were procured from Penta Manufacturing (Livingston, NJ, USA). All reagents and solvents were obtained from Fisher Scientific (Waltham, MA, USA) or Sigma Aldrich (St. Louis, MO, USA). Luria broth (LB) was purchased from Sigma Aldrich and yeast peptone dextrose (YPD) was prepared from Difco^TM^ yeast extract, Difco^TM^ Bacto peptone, and dextrose (all materials obtained from Fisher Scientific, Waltham, MA, USA) at a composition of 1% yeast extract, 2% peptone and 2% dextrose *w*/*v*.

YP loading of terpenes (terpene:YP *w*/*w* ratio of 1.1:1): Dry YPs were mixed with water (180 g YP/L) and the slurry was passed through an Emulsiflex^®^-C3 high pressure homogenizer (Avestin, Ottawa, ON, Canada) to obtain a uniform, single YP suspension. Samples of homogenized YP (8.35 g) were mixed with a geraniol-eugenol-thymol (GET) mixture (1.65 g GET at a composition of 2:1:2 GET weight ratio) and incubated at room temperature for a minimum of 24 h to allow for complete terpene loading by diffusion through the porous yeast cell wall into the hydrophobic hollow interior.

YP loading of terpenes (terpene: YP *w*/*w* ratio ≥ 2:1): Dry YPs were mixed with water for 30 min to obtain a uniform YP suspension (terpene loading at high terpene: YP ratios does not require homogenization of YPs) and then a 2:1:2 GET mixture was added to the YP sample and incubated at room temperature for a minimum of 24 h to allow for complete terpene loading. The amounts of YP, water, and terpene required to prepare YP GET with weight ratios of 1.1:1 to 5:1 are indicated in Table 3.

Characterization of terpene loading efficiency: Samples of YP-GET (10 µL, 10 mg YP/mL) were stained with Nile red (2 µL, 0.1 mg/mL) and fluorescein labeled concanavalin-A (FITC ConA, 2 µL, 0.1 mg/mL) to qualitatively assess loading by the fluorescence microscopy of the encapsulated fluorescent terpene-Nile red complex in the FITC ConA labeled yeast particle. Nile red was imaged using a rhodamine (red) filter (maximum excitation/emission wavelengths at 550/570 nm) and FITC-ConA was imaged with a green filter at 490/520 nm. Microscopy images were collected with an Olympus BX60 (Olympus, Waltham, MA, USA) upright compound fluorescent microscope [20]. YP GET samples (100 mg) were centrifuged to collect excess liquid (free terpene and water) and the pellet fraction was resuspended in 10 mL of 90% methanol–10% water to extract encapsulated terpenes. Terpenes were quantified by HPLC [20] operated with 32 Karat^TM^ software version 7.0 (Beckman Coulter, Inc, Brea, CA, USA), using a Waters Symmetry^®^ C18 column (3.5 µm, 4.6 × 150 mm) with acetonitrile:water 50:50 as mobile phase, flow rate at 1 mL/min, injection volume of 10 µL, and terpene detection at 254 nm. This isocratic HPLC method allows for the detection of the three terpenes in the GET mixture in a single run with the following retention times: 5.2 min (eugenol), 7.7 min (geraniol), and 9.8 min (thymol). The quantification of terpenes was done by measuring the peak area and interpolating the concentration using a calibration curve obtained with terpene standards.

Terpene release from YP-GET: YP-GET samples (100 mg) were suspended in water (10 mL) and incubated at room temperature for 24 h. Aliquots were collected at predetermined times, centrifuged, and the supernatant was collected to measure terpene released from the particles by HPLC. The initial YP-GET suspension in water (1.5 mg YP/mL) was diluted two-fold, incubated at room temperature for another 24 h, and samples were collected to quantify terpene release from YPs. Additional two-fold dilutions were done every 24 h until achieving >90% release from YPs.

YP-GET encapsulation storage stability: YP-GET samples (500 mg) were transferred into glass vials and stored at room temperature (23 °C) or 54 °C for two weeks. The third set of samples were subjected to three freeze (−20 °C)/thaw (23 °C) cycles. The encapsulation storage stability was assessed by Nile-red microscopy, yeast particle diameter measurement using ImageJ software [40], particle counting with a hemocytometer, and quantification of free and encapsulated terpene by HPLC before and after storage. 

Measurement of particle diameter: Microscopy images of YP control and YP-GET samples were obtained at 1000× *g* magnification. An image of a microscope calibration slide ruler was used to set the scale in pixels/μm in ImageJ software. The photomicrographs of YP samples were evaluated with the calibrated scale in ImageJ. The particle diameter along the major and minor axes of the YP ellipses was measured for 20 whole yeast cell particles per picture and a minimum of three pictures per sample.

Optimization of hyper-loaded YP-GET: Dry YP was mixed with water and glycerin (70% water, 30% glycerin) for 30 min to obtain a uniform YP suspension, and then 2:1:2 GET was added to the YP sample and incubated at room temperature for a minimum of 24 h to allow for complete terpene loading. YP-GET samples in water (control) and water-glycerin at YP concentration of 100 g YP/L and GET:YP ratios from 3:1 up to 4.5:1 were prepared, characterized for terpene encapsulation efficiency, and evaluated for encapsulation storage stability as described above.

Antimicrobial activity assays against model bacterial and fungal organisms: The antimicrobial activity of YP-GET was evaluated using a modified published microplate assay procedure [41]. Samples of YP-GET were suspended in 100 µL of growth medium (LB was used in antibacterial assays and YPD in antifungal assays) and added to the first row (Row A) of a 96-well plate (all wells in the 96-well plate contain additional 100 µL medium). Serial dilutions (1:1) were performed by transferring 100 µL from Row A to Row B, etc., and finally removing 100 µL from Row H. Diluted *Escherichia coli* Top10 (Invitrogen, Carlsbad, CA, USA), *Staphylococcus aureus* ATCC 19636, or *Candida albicans* SC5134 [42] cells (100 µL, 10^6^ cells/mL) was added to all wells of the plate. Initial (t = 0) and final (t = 16 h, 37 °C) absorbance readings were taken at 650 nm. The minimum inhibitory concentration (MIC) was determined as the concentration of terpene that inhibits bacterial or fungal growth as measured by absorbance by more than 75%.

Statistical Analysis: All experiments were conducted with a minimum of three replicates and the reported data correspond to average values with standard deviation. Prizm v. 9 was used for all graphs and two-group comparisons. For all comparisons, a two-tailed Student’s *t*-test was used.

## 4. Conclusions

Yeast particles can be used for the encapsulation of terpenes with high terpene loading capacity, up to a 5:1 terpene: YP ratio. A mixture of geraniol, eugenol, and thymol (GET) previously used to develop YP-GET 1.1:1 was used as a model terpene composition to prepare hyper-loaded YP-GET. The hyper-loaded YP-GET samples were produced with >95% GET encapsulation efficiency and the increased loading capacity, up to a 5:1 GET:YP ratio, is possible due to the high swelling properties of YPs allowing for an increase in YP diameter to accommodate the large terpene droplet at higher payload levels in the swollen hollow cavity of the particles. These hyper-loaded YP-GET samples enhanced sustained GET release up to three-fold compared to the previously developed YP-GET 1.1:1 formulation, show higher antimicrobial potency than unencapsulated GET, and improved thermal terpene storage encapsulation stability. Hyper-loaded YP-terpenes possess properties of interest for the development of YP terpene materials with a wide range of potential agricultural and pharmaceutical applications. Similarly, high levels of some hydrophobic actives can also be loaded into YPs, extending the versatility of the YP delivery technology.

## 5. Patents

Hyperloaded Yeast Cell Wall Particle and Uses Thereof, G.R. Ostroff, E.R. Soto and F. Rus. US Patent App. 63/346,012. 26 May 2022.

## Figures and Tables

**Table 2:** In vitro antimicrobial activity of negative YP control, YP-GET, unencapsulated GET, and positive drug controls (ampicillin and fluconazole) on two bacteria and one fungal model organism. The MIC results (mg/mL) represent the average of three biological replicate experiments with three technical replicates for each experiment. Statistically significant results were obtained between YP-GET 1.1:1 and unencapsulated GET for the tested microbes (* *p* < 0.1, *** *p* < 0.001, **** *p* < 0.0001) and between YP GET (all ratios) and ampicillin or fluconazole.

Sample	Minimum Inhibitory Concentration (MIC 75%)		
	*E. coli*	*S. aureus*	*C. albicans*
Empty YPs	Not active	Not active	Not active
YP-GET 1.1:1	0.156 ± 0.05	0.313 ± 0	0.156 ± 0.026
YP-GET 2:1	0.156 ± 0.06	0.313 ± 0	0.156 ± 0.026
YP-GET 3:1	0.156 ± 0.05	0.313 ± 0	0.156 ± 0.026
YP-GET 4:1	0.156 ± 0.03	0.313 ± 0	0.156 ± 0.039
YP-GET 5:1	0.156 ± 0.05	0.313 ± 0	0.156 ± 0.030
Unencapsulated GET	0.625 ± 0 ****	1.250 ± 0.318 *	0.625 ± 0 ***
Ampicillin	0.008 ****	<0.00025 ****	-
Fluconazole	-	-	<2 ****

**Table 3:** Yeast particle, water, and 2:1:2 GET quantities required to produce 100 g of YP GET at five GET:YP ratios at a constant YP concentration.

GET:YP Ratio	Materials to Produce 100 g YP-GET			Final Composition	
	g YP	mL (g) Water	g GET	%YP	%GET
1.1:1	15	68.5	16.5	15	16.5
2:1	15	55	30	15	30
3:1	15	40	45	15	45
4:1	15	25	60	15	60
5:1	15	10	75	15	75

## Data Availability

Data is contained within the article or Supplementary Materials.

## Supplementary Materials

The following supporting information can be downloaded at: https://www.mdpi.com/article/10.3390/molecules27113580/s1, Figure S1: Kinetics of 2:1:2 GET loading in YPs showing similar rate of absorption for each terpene of the 2:1:2 GET mixture, Figure S2: Cumulative GET release from YP GET 1.1:1 and YP GET 3:1 formulations in water and 0.9% saline, and Figure S3: Kinetics of terpene release showing similar release pattern for geraniol, eugenol, and thymol from YP GET formulations.

## Sample Availability

Samples of YP-GET are available from the authors upon request.
