# Supplementary material for: Yeast Particles Hyper-Loaded with Terpenes for Biocide Applications
Source: Molecules. 2022 Jun 2;27(11):3580. doi: 10.3390/molecules27113580 (PMC9182042; doi:10.3390/molecules27113580)
Supplement: Supplementary file 1 [file molecules-27-03580-s001.zip › molecules-1611754-supplementary.pdf]

Yeast Particles Hyper-loaded with Terpenes for Biocide Applications

Ernesto R. Soto, Florentina Rus, Gary R. Ostroff

Supplementary Materials

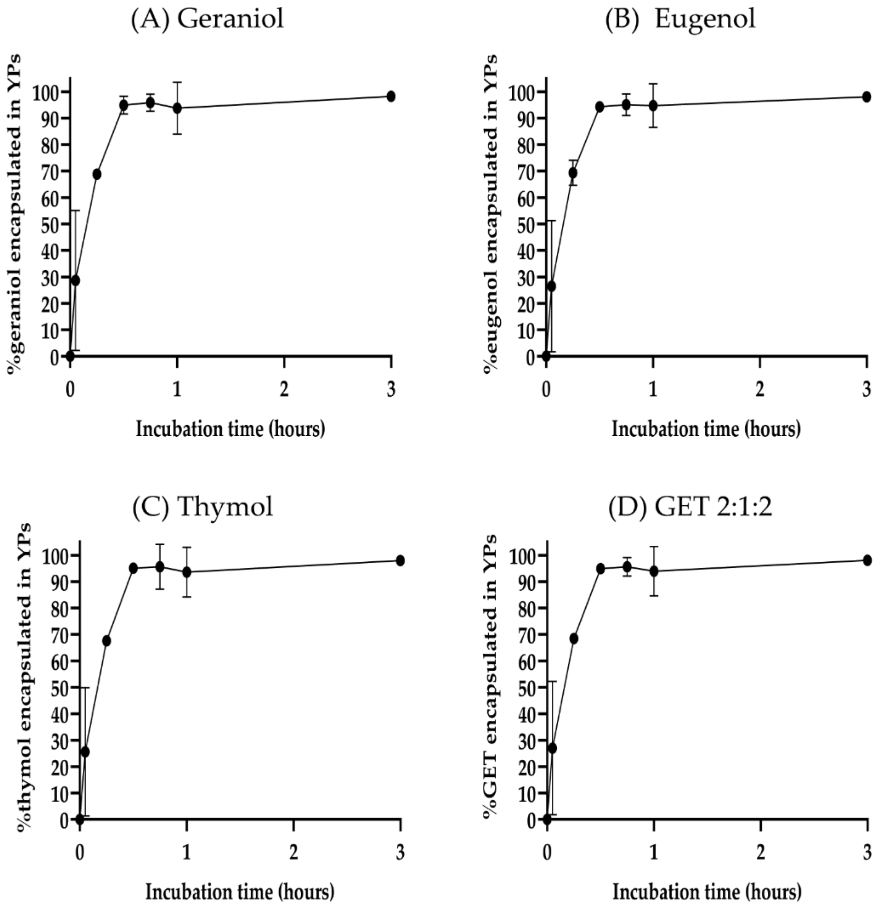

Figure S1. Kinetics of 2:1:2 GET loading in YPs showing similar rate of absorption for each terpene of the 2:1:2 GET mixture: (A) geraniol, (B) eugenol, (C) thymol, and (D) combined data of GET 2:1:2

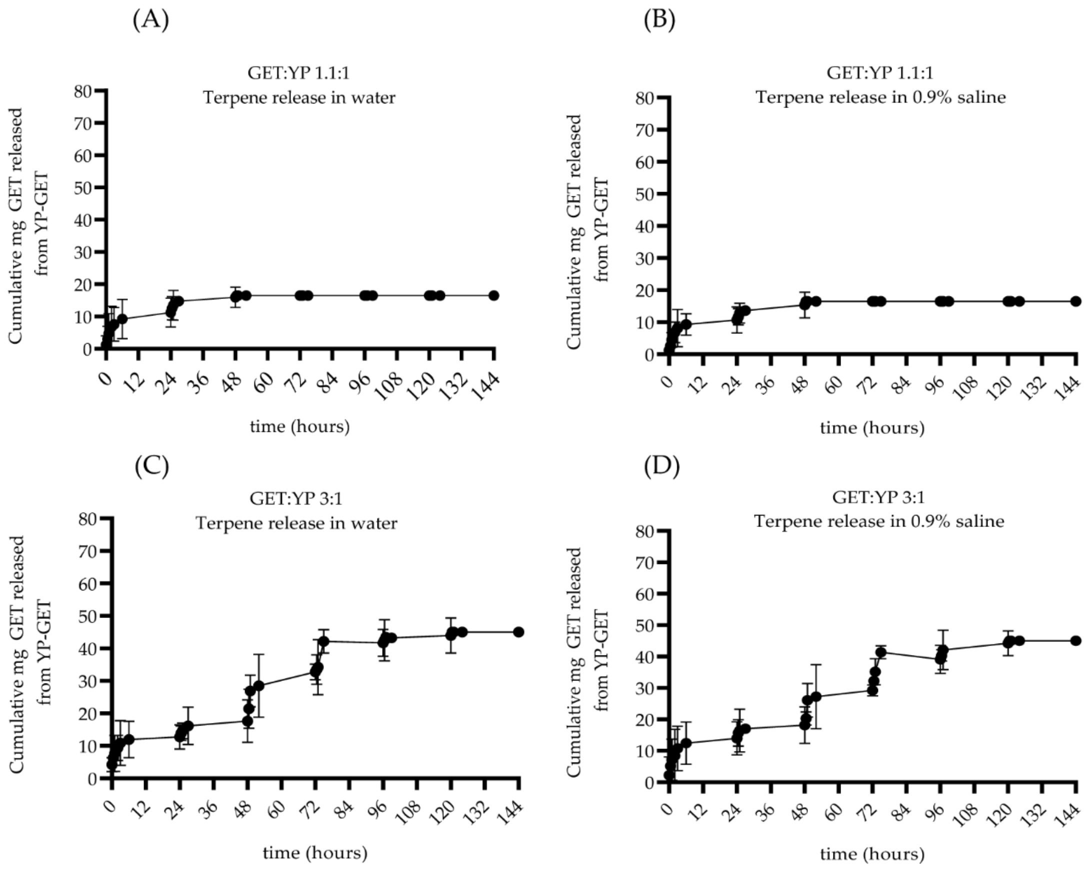

Figure S2. Cumulative GET release from YP GET 1.1:1 in (A) water, (B) 0.9% saline and YP GET 3:1 in (C) water and (D) 0.9% saline

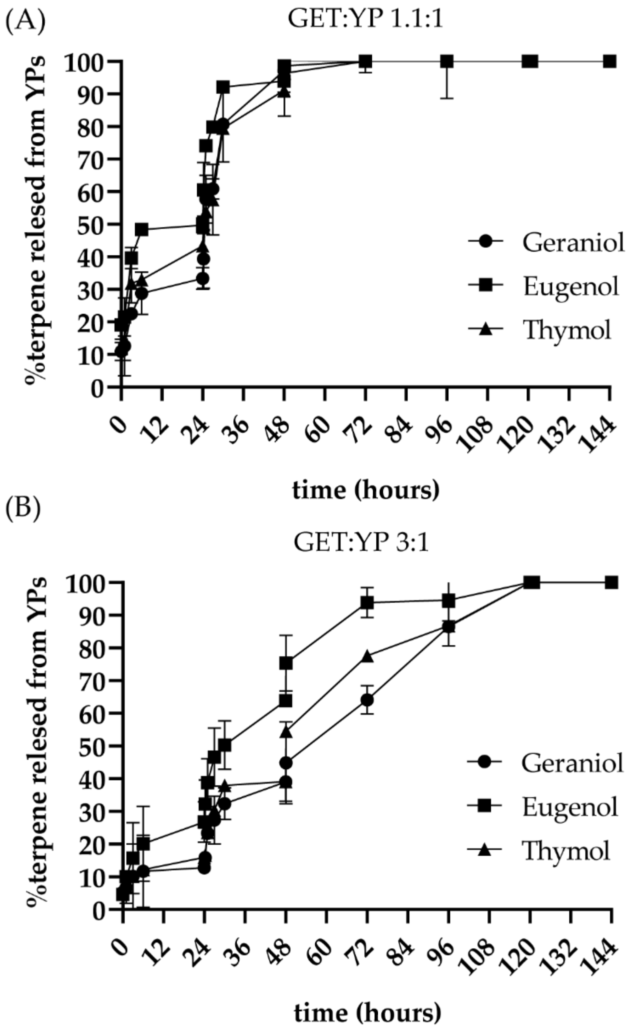

Figure S3. Kinetics of terpene release showing similar release pattern for geraniol, eugenol and thymol from YP GET formulations: (A) GET:YP 1.1:1, (B) GET:YP 3:1
